# Specific Activation of the Expression of Growth Factor Genes in Expi293F Human Cells Using CRISPR/Cas9-SAM Technology Increases Their Proliferation

**DOI:** 10.32607/actanaturae.27415

**Published:** 2024

**Authors:** P. A. Bobrovsky, E. N. Grafskaia, D. D. Kharlampieva, V. A. Manuvera, V. N. Lazarev

**Affiliations:** Lopukhin Federal Research and Clinical Center of Physical-Chemical Medicine of Federal Medical Biological Agency, Moscow,119435 Russian Federation; Moscow Institute of Physics and Technology, Moscow, 141701 Russian Federation

**Keywords:** CRISPR/Cas9-SAM, HEK293, proliferation, IGF-1, FGF-2, EIF3I

## Abstract

Human cell lines play an important role in biotechnology and pharmacology. For them to grow, they need complex nutrient media containing signaling proteins — growth factors. We have tested a new approach that reduces the need of cultured human cell lines for exogenous growth factors. This approach is based on the generation of a modified cell with a selectively activated gene expression of one of the endogenous growth factors: IGF-1, FGF-2, or EIF3I. We modified the Expi293F cell line, a HEK293 cell line variant widely used in the production of recombinant proteins. Gene expression of the selected growth factors in these cells was activated using CRISPR/Cas9 technology with the synergistic activation mediators CRISPR/Cas9-SAM, which increased the expression of the selected genes at both mRNA and protein levels. Upon culturing under standard conditions, the modified lines exhibited increased proliferation. A synergistic effect was observed in co-culture of the three modified lines. In our opinion, these results indicate that this approach is promising for efficient modification of cell lines used in biotechnology

## INTRODUCTION

Mammalian, in particular human, cell lines are of great importance in pharmacology, biotechnology, and basic research. Immortalized cell lines are cultured *in vitro *like microorganisms, but at the molecular level, they retain virtually all the features of the cells of the organism from which they are derived. However, unlike bacteria and yeast, mammalian cell lines are much more sensitive to culture conditions and nutrient medium composition. Apart from low-molecular compounds, these media should contain special signaling proteins that ensure cell proliferation, as a necessity. Upon routine culturing, these components are added to the medium, together with fetal bovine serum (FBS) that has variable composition and is expensive. Furthermore, the use of serum is unacceptable in the production of recombinant proteins for medical purposes, given low replicability of the results obtained and the existing restrictions on the use of components of animal origin [1, 2]. In addition, FBS negatively affects suspension culture, which requires the introduction of additional components or transition to adhesion culture and increases the cost of production by orders of magnitude [3]. The proteins necessary for normal cell growth can be added to the nutrient medium or produced by the cells. Because the cost of media and growth factors constitutes the bulk of the costs when culturing eukaryotic cells, the transition to culture in basic media can significantly reduce the cost of producing recombinant proteins

More than 70% of the recombinant proteins produced in eukaryotic expression systems are harvested in cultures of Chinese hamster ovary (CHO) cells [5]. Despite the fact that this cell line exhibits good proliferative activity and can produce recombinant proteins in large quantities, there are some limitations to its use. Some post-translational modifications of the proteins in CHO cells, such as the glycosylation pattern, are not typical of human proteins [6]. This circumstance may potentially render CHO-derived products immunogenic to humans [7]. One of the alternatives to CHO cells is the human HEK293 cell line. This line is better suited to the production of biotherapeutic drugs with post-translational modifications typical of human proteins. The HEK293 cell line, despite its epithelial origin and adhesive nature, has been adapted to suspension culture in serum-free or chemically defined media and is used to produce recombinant proteins both in laboratories and on an industrial scale [8]. Studies on the optimization of nutrient media have led to the development of several commercial formulations of chemically defined media and various additives in the form of animal-derived components, which have made possible the production of recombinant proteins in HEK293 cells in large quantities [9]. However, this cell line continues to lag behind the CHO line, which is a leader in the production of pharmaceutical recombinant proteins. HEK293 is inferior in proliferative activity, cultivation time, and product yield [10]. Activation of the expression of key growth factor genes in HEK293 cells may potentially increase their proliferative activity and productivity. The production of endogenous growth factors may help avoid the use of culture media containing components of animal origin and significantly reduce the cost of production compared to the use of commercial media containing purified growth factors. It should be noted that the use of recombinant growth factors as an additive to imitate the blood serum composition makes culture media very expensive, in particular due to the rapid degradation of the factors in the culture medium [11].

Given the abovementioned considerations, we set out to generate HEK293 cell lines with activated expression of one of three genes encoding the IGF-1, FGF-2, and EIF3I growth factors of cells. Positive effects of increased production of these growth factors on cell culture proliferation or the production of target recombinant proteins have been reported [12, 13, 14, 15]. The Expi293F line, a suspension HEK293 variant adapted to efficient production of recombinant proteins, was selected for modifications [16]. Expression of growth factor genes was activated using the synergistic activation mediator technology (CRISPR/Cas9-SAM), which is a variant of the CRISPR/Cas9 genome editing system. This system enhances the expression of target genes [17]. The proposed approach, on the one hand, enables very effective achievement of high expression levels of the target genes [18], and, on the other hand, selection of several options for fine-tuning the expression of growth factor genes. In addition, the use of the CRISPR/Cas9-SAM technology enables rapid activation of the expression of other growth factors and an analysis of their impact on the ability of a culture to grow in a basic serum-free nutrient medium. We demonstrate an activation of the expression of selected genes in the produced cell lines at both the mRNA and protein levels. Activation of the expression of growth factor genes increased the proliferation of the modified cell lines. The results obtained confirm the efficiency of the chosen approach in the generation of new human cell lines that can be used in biopharmaceuticals.

## EXPERIMENTAL

**Bacterial strains and cell lines**

The *E. coli *Top10 strain (Invitrogen, USA), the* F– mcrA Δ(mrr-hsdRMS-mcrBC) φ80lacZΔM15 ΔlacX74 nupG recA1 araD139 Δ(ara-leu)7697 galE15 galK16 rpsL(StrR) endA1 λ– *genotype, was used in the genetic engineering procedures.

The Expi293FTM (Gibco, USA) and Phoenix-AMPHO (ATCC CRL-3213) cell lines were used in thise study.

**Cell line cultivation**

For manipulations, the Expi293F cells were placed in adhesive growth conditions. The cells were cultured in DMEM (HiMedia, India) supplemented with 10% FBS (Gibco) and 1.5 μg/mL gentamicin (Gibco) in a CO_2_ incubator (Heraeus, Germany) at 37°C, relative humidity ≥80%, and 5% CO_2_.

**Construction of plasmids for producing lentiviral particles**

The CRISPR/Cas9-SAM expression activation system requires three lentiviral vectors that are produced using three plasmids: lenti_sgRNA(MS2)_ puro (Addgene #73795), lenti_MS2-P65-HSF1_Hygro (Addgene #61426), and lenti_dCAS-VP64_Blast (Addgene #61425) [17]. Two of these plasmids are used without further manipulation, and the lenti_ sgRNA(MS2)_puro plasmid is designed to carry a short DNA fragment encoding a single-guide RNA (sgRNA) protospacer. Before protospacer sequences were selected, the DNA segments corresponding to the 5’-adjacent regions of the growth factor genes were validated by sequencing. Protospacer sequences were selected using the CHOPCHOP service (https://chopchop.cbu.uib.no/). Six protospacer sequences were selected for the promoter region of each growth factor gene (IGF-1, FGF-2, EIF3I). To produce a vector encoding a chimeric sgRNA, the lenti_sgRNA(MS2)_puro vector was treated with the BsmBI restriction endonuclease (Thermo Fisher Scientific, USA) and then ligated with an oligonucleotide duplex corresponding to one of the protospacer sequences (Appendix 1, Appendix 2). Ligation products were cloned into the *E. coli *Top10 strain, and colonies carrying the target construct were selected. In total, we constructed 18 plasmid vectors based on lenti_sgRNA(MS2)_puro, which encoded sgRNAs targeting the promoter regions of the growth factor genes.

**Lentivirus production and transduction**

For lentivirus assembly, the culture medium of Phoenix-AMPHO cells was replaced with DMEM containing 25 μM chloroquine diphosphate (Sigma, USA) and incubated at 37°C and 5% CO_2_ for 5 h. Phoenix-AMPHO cells were transfected simultaneously with four (lenti_dCAS-VP64_Blast/lenti_ MS2-P65-HSF1_Hygro/lenti_sgRNA(MS2)_puro/ LeGo_G2 (Addgene #25917) [19], pMD2.G (Addgene #12259), and pRSV-Rev, pMDL/pRRE [20]) plasmids using polyethyleneimine: PEI MAX 40K (1 mg/mL, Polysciences, USA) (DNA:PEI ratio = 1:3) according to the previously described technique [21]. After incubation at room temperature for 20 min, the DNA–PEI mixture was added dropwise to the cells and incubated for 6 h. The medium was then changed to Opti-MEM containing 2 mM sodium butyrate (Sigma). After 48 h, the medium containing lentiviruses was filtered through a 0.22-μm filter (TPP, Switzerland), added with 100 μg/mL protamine sulfate (Ellara, Russia), and immediately applied to Expi293F cells for infection. After incubation for 24 h, the medium was replaced with a fresh, complete DMEM medium. After lentiviral transduction, the cells were passaged at least 3 times with an appropriate antibiotic. For lenti_dCAS-VP64-Blast, lenti_MS2-P65-HSF1-Hygro, and lenti_gRNA-puro lentiviruses, we used blasticidin at a concentration of 7 μg/mL, hygromycin at a concentration of 300 μg/mL, and puromycin at a concentration of 2 μg/mL, respectively. To determine the transduction efficiency in the cells after three passages, the expression of the target gene was analyzed.

**Quantitative PCR**

Transduced cells were removed using 0.05% trypsin with EDTA (Gibco), centrifuged at 500 g for 5 min, and the supernatant was collected. Total RNA was isolated from samples containing approximately 106 cells using the Trizol reagent (Thermo Fisher Scientific). Total RNA was treated with 2 enzyme units of DNase I (Thermo Fisher Scientific) in the presence of 20 enzyme units of the ribonuclease inhibitor (Thermo Fisher Scientific). cDNA was synthesized using a RevertAid RT Reverse Transcription kit (ThermoFisher Scientific) with hexamer primers. Real-time PCR was performed on a CFX96 Touch amplifier (BioRad, USA) using a 5X qPCRmix-HS SYBR ready-to-use PCR mixture (Evrogen, Russia). Data were normalized to the *gapdh *reference gene level (Appendix 1).

**Measurement of proliferative activity**

To visualize an increase in modified cells, 2 × 10^4^ cells were seeded per well of a six-well plate, cultured in DMEM containing 10% FBS in a carbon dioxide incubator at 37°C and 5% CO_2_ for 24 h, then transferred to a Celena X High Content Imaging System (Logos Biosystems, Republic of Korea) for live cell imaging, and cultured in an isolated chamber at 37°C and 5% CO_2_ for 190 h. As evaporation occurred, the wells were replenished with a nutrient medium. The cells in the GFP channel (470/530 nm) were imaged at 10× magnification every 8 h, 324 fields of view per well, with laser autofocus every 10 fields of view. The images were processed using the Celena EXPLORER software (Logos Biosystems).

To compare the growth of the modified cells in a basic medium free of FBS, we measured the electrical resistance (impedance) between electrodes located at the bottom of the plate wells using an xCELLigence RTCA DP biosensor cell analyzer (Agilent, USA). Changes in impedance depend on the contact area between the cells and an the electrode; this parameter is used to automatically calculate the cell index that characterizes the status of the cell culture at a given time. Modified cells were seeded at 104 cells per well of a 16-well E-plate (Agilent). After 40 h, the medium was replaced with a fresh DMEM medium containing 1.5 μg/mL gentamicin, with/without 10% FBS. The plates were transferred to the cell analyzer, and the cells were cultured at 37°C and 5% CO_2_ for 120 h. Electrical resistance was measured every 30 min.

The effect of a conditioned medium on the growth of unmodified cells was compared using a colorimetric test with tetrazolium salt. Expi293F cells were seeded at 104 per well of a 96-well plate. After 24 h, 100 μL of the conditioned medium from modified cells was added to each well. To produce the conditioned medium, a monolayer of modified cells was washed with PBS and incubated with the Opti-MEM medium (Gibco) for 48 h. The conditioned medium from Expi293F-dCas9-MS2 cells was used as a control. The medium was collected and concentrated 10-fold using a Microcon 3 kDa centrifugal filter (Millipore, USA). The conditioned medium was mixed with DMEM or the Opti-MEM basic medium containing 1.5 μg/mL gentamicin, an amino acid solution (Himedia), a vitamin solution for RPMI 1640 (Himedia) at the 1 : 10 ratio, and used in the experiments. After 96 h, the cells were added with an MTT solution to a final concentration of 5 μg/mL. the plate was incubated in an incubator for 4 h. A solubilizing solution was added, and absorbance was measured at 570/690 nm on a Multiscan Ascent microplate reader (Thermo Fisher Scientific).

**Immunoblot**

For Western blot hybridization, the proteins separated during electrophoresis were transferred to a 0.45 μm PVDF membrane (Amersham Biosciences, USA). The transfer was performed in a Hoefer TE77XP chamber (USA) at a current of 0.8 mA/cm2 and a voltage limited to 30 V for 1 h. To detect IGF-1, the PAA050Hu06 antibody (CloudClone, USA) was used at a dilution of 1 : 1,000; for FGF-2, the PAA551Hu01 antibody (CloudClone) was used at a dilution of 1 : 400; and for EIF3I, the DF12393 antibody (Affinity Biosciences, China) was used at a dilution of 1 : 2,000.

**Statistical analysis**

The statistical analysis was performed using the Mann–Whitney U test and the Python programming language (version 3.12) (Python Software Foundation, USA).

## RESULTS

**Generation of an Expi293F intermediate cell line carrying the common components of the CRISPR/Cas9-SAM system**

Three components are required to activate gene expression in the CRISPR/Cas9-SAM system. In this regard, lentiviral particles carrying an integration cassette encoding the dCas9-VP64 fusion protein gene, particles encoding the MS2-p65-HSF chimeric protein, and particles encoding sgRNA were produced in Phoenix-AMPHO cells. Expi293F cells were transduced with the lentiviral particles. Transduction using the MS2-P65-HSF1_Hygro vector resulted in the Expi-MS2 precursor line carrying a construct encoding the chimeric MS2-p65-HSF protein. After selection on a hygromycin-containing medium and cloning, a line with high expression of the recombinant* MS2-P65-HSF1 *gene was selected using quantitative RT-PCR. Then, the Expi-MS2 line was transduced with dCas9-VP64 lentiviral particles to produce the Expi-dCas9-MS2 precursor line encoding the chimeric MS2-p65-HSF protein and the defective dCas9-VP64 nuclease. After cell transduction, selection was performed on a medium containing hygromycin and blasticidin. Expression of recombinant genes was confirmed by quantitative RT-PCR. Therefore, we succeeded in generating an Expi293F precursor line encoding two components of the CRISPR/Cas9-SAM system and suitable for transduction with lentiviral vectors encoding specific sgRNA.

**Generation of cell lines with activated expression of growth factor genes**

**Fig. 1:**
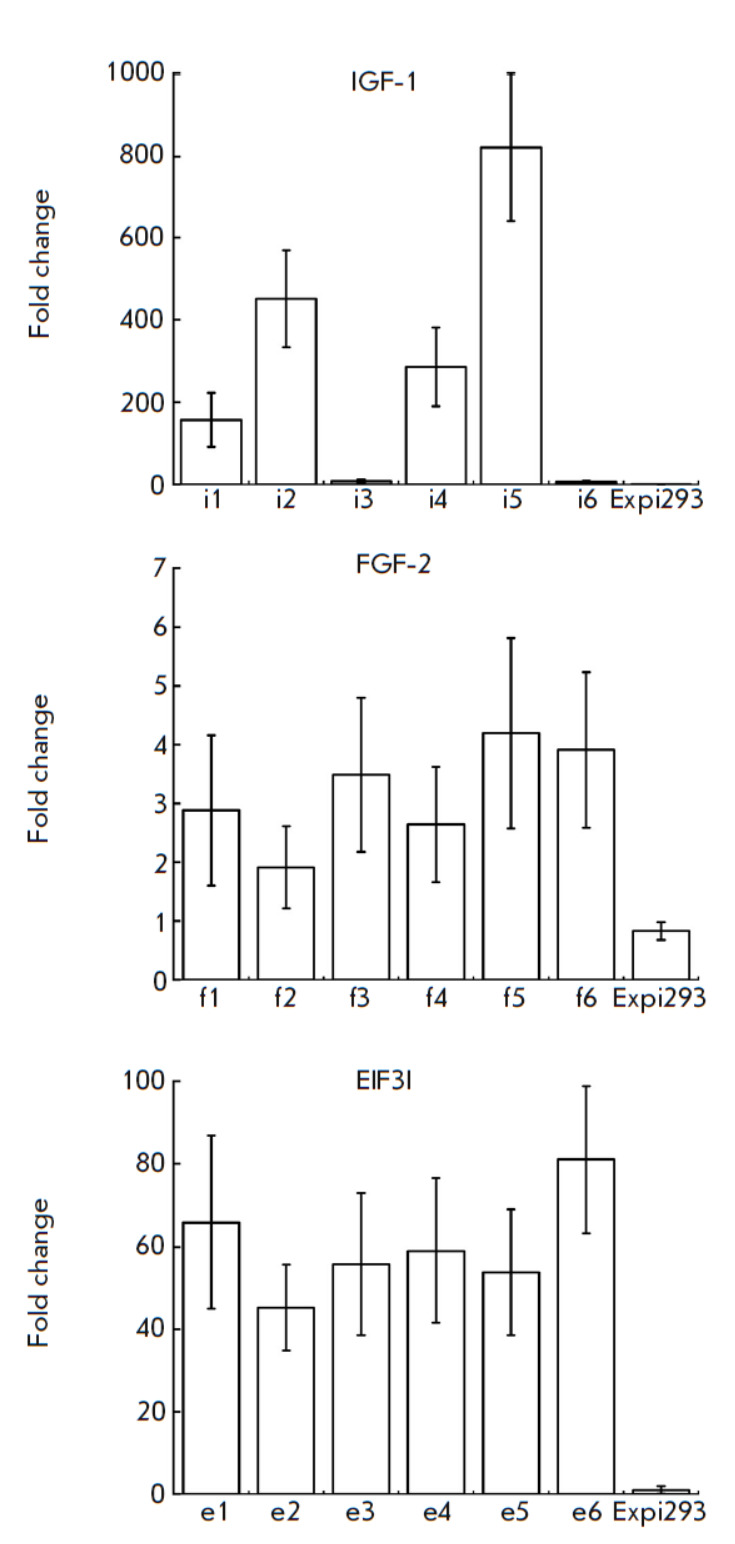
Fold change of IGF1, FGF2, and EIF3I genes in Expi-dCas9-MS2 cell lines transduced with lentiviral vectors encoding sgRNAs targeting the promoter regions of the respective genes. Lentiviral vectors encoding i1–i6, f1–f6, and e1–e6 sgRNAs (Appendix 2) were used to activate* IGF1*, *FGF2*, and *EIF3I *gene expression. IGF1 expression is not typical of HEK cells; so, fold change was calculated relative to the lowest IGF1 expression in the modified cell line (Expi-dCas9-MS2-i6). FGF2 and EIF3I are expressed in human embryonic kidney cells; so, changes in the expression levels were evaluated relative to the Expi-dCas9-MS2 cell lines. Data are presented as a mean ± SEM

For targeted activation of the expression of one of the four selected genes, Expi-dCas9-MS2 cells were transduced with lentiviral particles encoding sgRNA. As described above, we constructed six vectors for each of the selected growth factors and produced lentiviral particles. The vectors were named using the first letter of the gene name and the serial number of the protospacer sequence (Appendix 2). Next, we selected the most promising sgRNAs, which provided increased expression of genes of the IGF-1, FGF-2, and EIF3I growth factors. After transduction, the cells were cultured for 72 h. Transcriptional activation of growth factor genes was determined by quantitative RT-PCR (*Fig. 1*).

**Table 1:** Protospacer sequences of guide RNAs that ensure the highest level of target gene expression

Gene	Protospacer	Construct
IGF-1	AGGCATACAATGGAAATAGG	i2
IGF-1	GTGTTTTGTAGATAAATGTG	i5
FGF-2	GGCCGAACCGCCGAACTCAG	f5
FGF-2	CGCGCGACATCAGTCCGGCG	f6
EIF3I	AGGATCCTTCCAGGGCAAAG	e1
EIF3I	GAATGTCTTTCCTTGGAGGG	e6

*IGF-1* gene expression was observed for four out of six sgRNAs. Since *IGF-1* is not expressed in human embryonic kidney cells, it is impossible to assess any changes in its expression. In further study, we investigated the lines generated using i2 and i5 sgRNAs because analysis of these lines by real-time PCR revealed the lowest threshold cycle value (*Table 1*). Analysis of the *FGF-2 *gene expression level showed that expression of all sgRNAs enhanced *FGF-2 *expression 2- to 5-fold. f5 and f6 sgRNAs were selected for further work (*Table 1*). Also, expression of all six sgRNAs was shown to increase the expression of the EIF3I factor gene 44- to 81-fold. Then, we studied the lines produced using e1 and e6 sgRNAs (*Table 1*).

**Detection of induced accumulation of growth factors using immunoblotting**

**Fig. 2:**
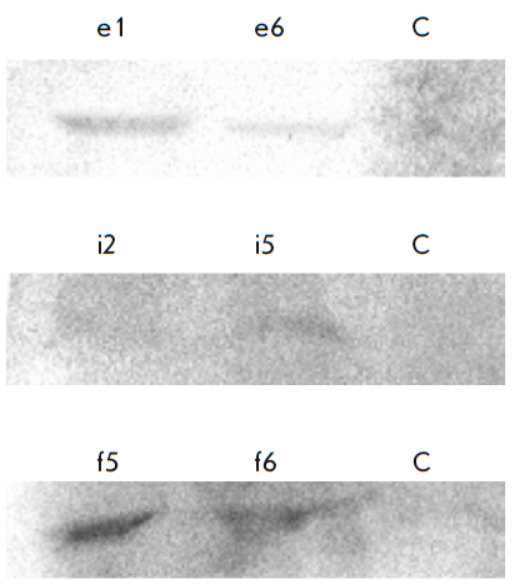
Western blot analysis of culture medium samples from Expi293F cells with activated expression of growth factor genes. e1 and e6 samples are cells with activated expression of the *EIF3I *gene; f5 and f6 samples are cells with activated expression of the *FGF2 *gene; i2 and i5 samples are cells with activated expression of the *IGF1* gene. C is Expi293F cells

The expression products of the activated genes were detected by Western blot hybridization using antibodies specific to the growth factors under study. In the case of the secreted growth factors (IGF-1, FGF-2), we analyzed the culture medium; to detect the intracellular growth factor (EIF3I), we analyzed the cell lysates (*Fig. 2*).

The analysis revealed the EIF3, IGF1, and FGF2 factors in the culture medium of the cells transduced with lentiviral vectors encoding e1, e6, i2, i5, f5, and f6 sgRNAs (*Table 1*).

**Proliferation of modified cell lines**

**Fig. 3:**
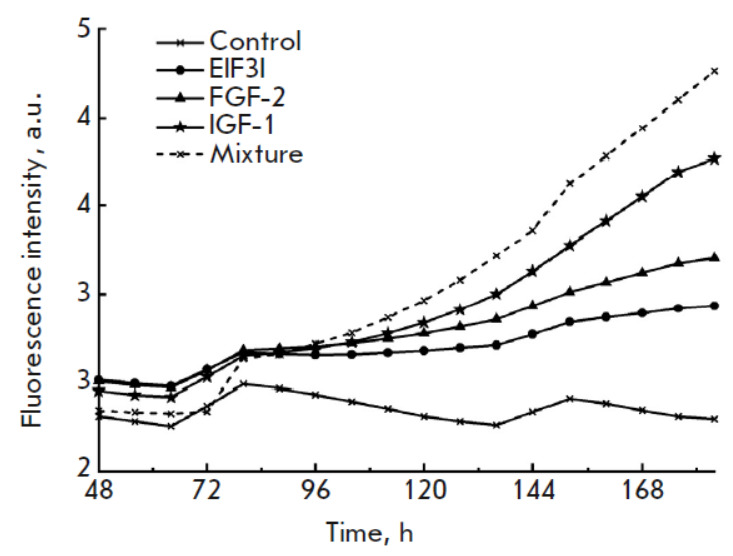
Proliferative activity of modified cell lines. Expi293F-dCas9-MS2 (GFP) cells cultured in a DMEM medium containing 10% FBS were transduced with lentiviral vectors encoding sgRNAs to activate the expression of growth factor genes. The fluorescence intensity was measured using the Celena X High Content Imaging System. The total fluorescence intensity in each field of view was assessed using the FIJI software [22]. The control is Expi293F-dCas9-MS2 (GFP) cells. EIF3I, FGF-2, and IGF-1 are cells with increased expression of the corresponding growth factor genes, which were produced by transduction with lentiviral vectors encoding e6, f6, and i2 sgRNAs, respectively. The mixture is a co-culture of Expi-IGF1, Expi-FGF2, and Expi-EIF3I cells

The proliferative activity of the cell lines was studied using the Celena X High Content Imaging System. To facilitate the visualization and subsequent data processing, the Expi293F-dCas9-MS2 cells were transduced with a lentiviral vector encoding the green fluorescent protein and then with the e1, e6, i2, i5, f5, and f6 lentiviral vectors to activate the expression of growth factor genes. Proliferation was assessed by analyzing changes in the fluorescence intensity of the cells due to the accumulation of the eGFP protein in them. In addition to cell lines expressing only one of the studied factors (*IGF-1*, *EIF3I*, and *FGF-2*), mixed culture of all three lines was also studied to assess the potential of a synergistic effect (*Fig. 3*).

**Fig. 4:**
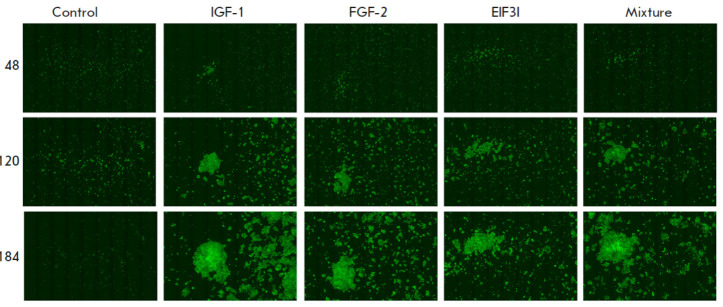
Comparison of the growth of modified cells expressing IGF-1, FGF-2, and EIF3I growth factor genes. The control is Expi293F-dCas9-MS2 cells transduced with the LeGo-G2 lentiviral vector. IGF-1, FGF-2, and EIF3I are Expi293F-dCas9-MS2 cells transduced with the LeGo-G2 lentiviral vector and the i2, f6, and e6 vectors, respectively. The mixture is a co-culture of Expi-IGF1, Expi-FGF2, and Expi-EIF3I cells. The images were acquired using the Celena X High Content Imaging System. Each image contains 72 fields of view

As a result, we showed increased proliferative activity of both cells with elevated expression of each of the IGF-1, EIF3I, and FGF-2 factors (*Fig. 4*) and cocultured cells. In this case, the proliferative activity was higher in co-cultured cells.

**Fig. 5:**
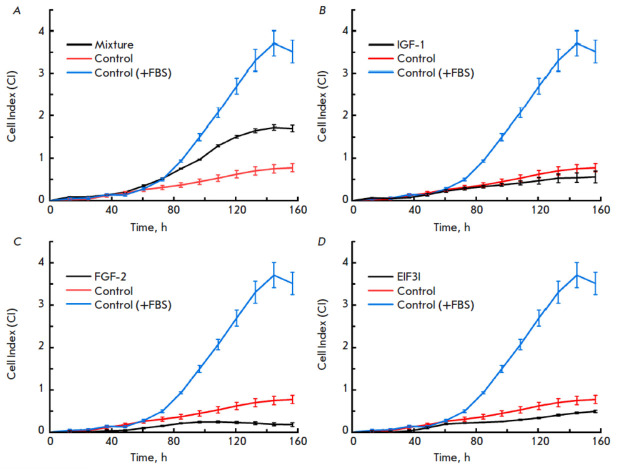
Analysis of modified cell proliferation in a DMEM basic serum-free medium using real-time impedance measurements. The control is Expi293F-dCas9-MS2 cells cultured in a DMEM medium. The control (+FBS) is Expi293F-dCas9- MS2 cells cultured in a DMEM medium supplemented with 10% FBS. The mixture (*A*) is a co-culture of Expi-IGF1, Expi-FGF2, and Expi-EIF3I cells. IGF1 (*B*), FGF2 (C), and EIF3I (*D*) are Expi293F-dCas9-MS2 cells transduced with the i2, f6, and e6 lentiviral vectors, respectively. Data are presented as a mean ± SEM for *n *= 4

Cell proliferation in the basic serum-free medium was studied using the xCelligence RTCA DP cell analyzer, which enables to assess the viability of cell cultures in real time without additional markers and labels. The proliferative activity of co-cultured cells expressing IGF-1, EIF3I, and FGF-2 in a FBS-free DMEM medium was shown to be higher than that of unmodified cells (*Fig. 5A*), but lower than that of un modified cells cultured in a medium containing 10% FBS. In cell cultures producing one of the studied factors, no increase in the proliferative activity was observed (*Fig. 5B-D*).

Thus, co-culture of modified cells expressing IGF-1, EIF3I, and FGF-2 in an FBS-free medium increased their proliferative activity compared with that in the control cells.

**Fig. 6:**
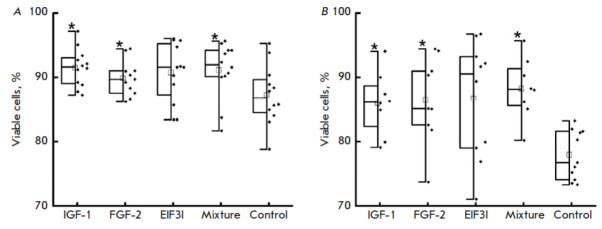
Viability assay of cells cultured in a conditioned medium from modified cells. IGF1, FGF2, EIF3, Mixture, and Control are conditioned media from the corresponding Expi293F-dCas9-MS2 cell lines transduced with the i2, f6, and e6 lentiviral vectors, respectively. A culture medium from non-modified Expi293F-dCas9-MS2 cells was used as the control. Data are presented as box-and-whisker plots, with the box indicating the 25^th^ and 75^th^ percentiles, the whiskers representing the interquartile range, the black line within the boxes representing the median, the square representing the mean, and the asterisk (*) indicating significant differences from the control at *p * < 0.05. Dots are viability values for each replicate. (*A*) Conditioned medium mixed with a Opti-MEM medium; (*B*) conditioned medium mixed with a DMEM medium free of FBS

**The effect of secreted growth factors on intact cell viability**

To assess the effect of secreted growth factors on the viability of intact cells, the conditioned medium was collected, filtered, and added into Expi293F cells. After 96 h, cell viability was measured using a colorimetric test (*Fig. 6*). The metabolic activity of the cells, in contrast to that of the control samples, was shown to increase after the addition of a conditioned medium from cells expressing individual factors (IGF-1, EIF3I, and FGF-2). The mixture of conditioned media also had a positive effect on the viability of intact cells. The difference in cell viability upon the use of Opti-MEM (*Fig. 6A*) or DMEM (*Fig. 6B*) was insignificant.

## DISCUSSION

For an efficient production of recombinant proteins for medical purposes, it is desirable to use human cell cultures capable of undergoing suspension culture in liquid media of a composition that is as simple as possible, ideally free of animal-derived components. A human cell culture is capable of generating proteins whose processing is as close as possible to that in the human body. Suspension culture enables the use of bioreactors for culture growth and relatively easy scaling of production. Simple medium composition ensures cost-effectiveness, and minimum use of animal-derived additives allows one to avoid contamination of the final product with undesirable impurities. In this study, we tested a fundamental approach that may produce such cell cultures for biotechnological purposes. Our approach is based on selective activation of the expression of endogenous genes encoding protein factors that enhance cell proliferation.

The HEK293 human embryonic kidney cell line is the human cell line most commonly used in biotechnology. We used an Expi293F cell line, a suspension variant of the HEK293 cell line, that is optimized for highly efficient production of recombinant proteins at high densities (the culture remains viable at a density of 5 × 10^6^ cells/mL) and a doubling of time of about 24 h [16]. Expi293F requires a number of growth factors for proliferation [23]. Our approach aims to activate the expression of the corresponding genes in the cells rather than add growth factors to the culture medium. Currently, a powerful tool for selective gene activation is available – the synergistic activation mediator technology (CRISPR/Cas9-SAM), which is a variant of the CRISPR/Cas9 genome editing system. It uses sgRNA that complementarily binds to a selected region of the promoter of the activated gene. As in genome editing, the Cas9 protein binds to the sgRNA. We used a mutant Cas9 protein lacking nuclease activity and fused with a VP64 tetramer, which enabled recruitment of transcription factors and activation of mRNA synthesis [24].

As the first target, we chose the genes of three factors: IGF-1, FGF-2, and EIF3I. Insulin is known to be necessary for cell proliferation in a serum-free medium [25] and is used in some modified basic media. Along with insulin, there are many insulin-like growth factors (IGFs) that also stimulate cell proliferation. IGF-1 and insulin belong to the same family and have similar tertiary structures. In addition, activation of* IGF-1 *gene expression by genomic editing has a positive effect on cell proliferation [26]. Fibroblast growth factors (FGF-1 and FGF-2) are important components of a medium for culturing cells, especially for maintaining their ability to proliferate [12]. These proteins have been produced in *Escherichia coli *and eukaryotic cells. In this case, it has been shown that FGF-1 and FGF-2 are prone to proteolytic degradation and denaturation in the cellular environment, which results in a relatively short half-life of these factors, and the recommended concentrations (10–100 ng/mL) complicate their use as medium additives [27, 28]. Cell culture proliferation is regulated by slowing down or accelerating transitions between different phases of the cell cycle. An increase in the growth rate due to overexpression of genes that promote the G1/S transition, such as eukaryotic initiation factor 3 (EIF3), enhances the production of recombinant proteins. EIF3 is a large multidomain protein, the individual subunits of which also exhibit functional activity [13]. EIF3I was earlier shown to be involved in an increasing proliferative activity of cells, as the expression of the gene for this factor was elevated and in decreasing activity in knockout was observed [29]. In addition, there is a report of an increase in the culture growth rate upon overexpression of genes, such as *eIF3I*, that promote the G1/S transition [30].

At the first step, we sequenced the promoter regions of the selected genes. Next, we generated a modified Expi293F cell line expressing the dCAS-VP64 and MS2-P65-HSF1 proteins required for the CRISPR/Cas9-SAM system to function [17]. Expression of the introduced genes at the RNA level was confirmed by quantitative RT-PCR. Then, we modified the produced Expi-dCas9-MS2 line by introducing one of the recombinant sgRNA-encoding genes into its genome. However, sgRNAs specific to different promoter regions may have different degrees of efficiency. Therefore, we selected six sequences of sgRNA protospacer regions for each target to compare their effect on expression activation. The comparison was performed using quantitative RT-PCR. Most likely, each of the growth factors has its own optimal expression level, but determining this level requires a separate, large study. Thus, we selected one sgRNA variant for each gene, which provided the highest activation of transcription.

For a number of reasons, transcription activation does not always lead to the accumulation of the corresponding protein. The presence of one of the three proteins in each of the three selected modified cell lines was confirmed by immunoblotting. The presence of the target proteins, the EIF3, IGF-1, and FGF-2 factors, was demonstrated. At this stage of the study, the presence of growth factors was assessed only qualitatively.

At the first stage of the investigation of modified cell proliferation, we used a DMEM basic nutrient medium supplemented with 10% FBS and the Celena X High Content Imaging System. This system provides real time micro-images of the culture plate surface. In this study, the Expi-IGF1, Expi-FGF2, and Expi-EIF3I cell lines were infected with lentiviral particles encoding the green fluorescent protein. Although the Celena X system enables one to conduct an analysis of cells in transmitted light, the use of fluorescent proteins facilitates the visualization of the cells under study and the total fluorescence intensity can be used as a quantitative indicator. We chose the lentiviral vector, because it ensures integration of the recombinant green fluorescent protein gene into the genomic DNA of the cell and its further transmission during cell division without the losses typical of transient transfection of plasmid DNA. All three modified cell lines turned out to grow faster than the control line (*Fig. 3*, *Fig. 4*). The most profound effect on proliferation was exerted by an increase in the expression of the IGF-1 factor gene. At the same time, co-culture of all three modified lines yielded a greater proliferative effect than that of each line taken individually. An increase in the proliferative activity of cells cultured in a basic serum-free medium was observed only upon co-culture of cells producing all three growth factors, but proliferation of these cells was lower than that upon culture in a medium containing FBS (*Fig. 5*). Enhanced cell growth in co-culture is quite expected, because the entire set of growth factors is required for cell proliferation in culture. Despite this, three factors are not enough to completely switch to the use of basic media free of FBS. In addition, without FBS, there is no increased proliferation in a culture of cells producing one of the factors (*Fig. 5B–D*). However, the contribution of individual factors is noticeable in the presence of FBS (*Fig. 3*, *Fig. 4*). FBS-based stimulation of the proliferation of cell lines expressing the EIF3I factor was described earlier [30].

We also analyzed the effect of a conditioned medium from modified lines incubated in a Opti-MEM medium for 48 h on the viability of Expi293F cells. This medium was chosen for initial culture, because it ensures good culture growth and is free of FBS, which may cancel the effect of the factors under study. A conditioned medium from modified cell lines increases the metabolic activity of intact cells compared with that of the control. The observed effect was related to both media from cell lines expressing one of the factors and a mixture of conditioned media.

Our findings suggest that careful selection of activated genes and control of their activation level may be used to generate a human cell line that is either less dependent on exogenous signaling proteins or even completely independent of them. The development of such a line will significantly reduce the cost of and simplify the production of recombinant proteins for medical purposes.

## CONCLUSION

By using the CRISPR/Cas9-SAM synergistic activation mediator technology, we generated an Expi293F human cell line with enhanced expression of the genes encoding the IGF-1, FGF-2, and EIF3I factors. Then, we demonstrated the activation of target gene expression both at the mRNA and protein levels. The modified cell lines exhibited increased proliferative activity under standard culture conditions and in co-culture of three producer lines in a DMEM basic medium free of FBS. In our opinion, our findings indicate that there is a possibility to use the selected approach in biotechnology. We believe that activation of various endogenous growth factor genes enables the production of cell lines that possess increased productivity and can grow on simple and inexpensive nutrient media.

## Appendix 1

**Table 1:** List of the oligonucleotides used in the study

Name	Sequence 5’>3’	Application
igf1_F	TGGGTTTTACAGCTCGGCAT	Sequencing
igf1_R	GGAAACAGCTGGGGGAACAT	Sequencing
fgf2_F	AAGCCTGCTCTGACACAGAC	Sequencing
fgf2_R	GTTCACGGATGGGTGTCTCC	Sequencing
eif3i_F	GGGATCCACACTGGTTGAGG	Sequencing
eif3i_R	TCACTCGTCTGCATTCAGGG	Sequencing
i1_F	CACCGTGTAGACAGGAAACAGCTGG	Constructing sgRNA(MS2)_puro-i1
i1_R	AAACCCAGCTGTTTCCTGTCTACAC	Constructing sgRNA(MS2)_puro-i1
i2_F	CACCGAGGCATACAATGGAAATAGG	Constructing sgRNA(MS2)_puro-i2
i2_R	AAACCCTATTTCCATTGTATGCCTC	Constructing sgRNA(MS2)_puro-i2
i3_F	CACCGTATTTCCAAGTGAGTGAGT	Constructing sgRNA(MS2)_puro-i3
i3_R	AAACACTCACTCACTTGGAAATACC	Constructing sgRNA(MS2)_puro-i3
i4_F	CACCGCACTAACACACATTCTTTTA	Constructing sgRNA(MS2)_puro-i4
i4_R	AAACTAAAAGAATGTGTGTTAGTGC	Constructing sgRNA(MS2)_puro-i4
i5_F	CACCGTGTTTTGTAGATAAATGTG	Constructing sgRNA(MS2)_puro-i5
i5_R	AAACCACATTTATCTACAAAACACC	Constructing sgRNA(MS2)_puro-i5
i6_F	CACCGCTCTAGTTTTAAAATGCAA	Constructing sgRNA(MS2)_puro-i6
i6_R	AAACTTGCATTTTAAAACTAGAGCC	Constructing sgRNA(MS2)_puro-i6
f1_F	CACCGATAAGGGGCGGTGGAGCCC	Constructing sgRNA(MS2)_puro-f1
f1_R	AAACGGGCTCCACCGCCCCTTATCC	Constructing sgRNA(MS2)_puro-f1
f2_F	CACCGGCAGGGCTTTGGCATTCCC	Constructing sgRNA(MS2)_puro-f2
f2_R	AAACGGGAATGCCAAAGCCCTGCCC	Constructing sgRNA(MS2)_puro-f2
f3_F	CACCGGGCCGGCCTCTGAGTTCGG	Constructing sgRNA(MS2)_puro-f3
f3_R	AAACCCGAACTCAGAGGCCGGCCCC	Constructing sgRNA(MS2)_puro-f3
f4_F	CACCGGAATGCCAAAGCCCTGCCG	Constructing sgRNA(MS2)_puro-f4
f4_R	AAACCGGCAGGGCTTTGGCATTCCC	Constructing sgRNA(MS2)_puro-f4
f5_F	CACCGGCCGAACCGCCGAACTCAG	Constructing sgRNA(MS2)_puro-f5
f5_R	AAACCTGAGTTCGGCGGTTCGGCCC	Constructing sgRNA(MS2)_puro-f5
f6_F	CACCGCGCGCGACATCAGTCCGGCG	Constructing sgRNA(MS2)_puro-f6
f6_R	AAACCGCCGGACTGATGTCGCGCGC	Constructing sgRNA(MS2)_puro-f6
e1_F	CACCGAGGATCCTTCCAGGGCAAAG	Constructing sgRNA(MS2)_puro-e1
e1_R	AAACCTTTGCCCTGGAAGGATCCTC	Constructing sgRNA(MS2)_puro-e1
e2_F	CACCGACATTTGTGAGCCTTTCCAG	Constructing sgRNA(MS2)_puro-e2
e2_R	AAACCTGGAAAGGCTCACAAATGTC	Constructing sgRNA(MS2)_puro-e2
e3_F	CACCGTGCAGGATGGGAACTAGCAG	Constructing sgRNA(MS2)_puro-e3
e3_R	AAACCTGCTAGTTCCCATCCTGCAC	Constructing sgRNA(MS2)_puro-e3
e4_F	CACCGATGAATTCGAGGCGAGGGTC	Constructing sgRNA(MS2)_puro-e4
e4_R	AAACGACCCTCGCCTCGAATTCATC	Constructing sgRNA(MS2)_puro-e4
e5_F	CACCGTGGGGTTGTAGCAGGGGTCG	Constructing sgRNA(MS2)_puro-e5
e5_R	AAACCGACCCCTGCTACAACCCCAC	Constructing sgRNA(MS2)_puro-e5
e6_F	CACCGAATGTCTTTCCTTGGAGGG	Constructing sgRNA(MS2)_puro-e6
e6_R	AAACCCCTCCAAGGAAAGACATTCC	Constructing sgRNA(MS2)_puro-e6
q_igf1_F	CTCTTCAGTTCGTGTGTGGAGAC	qPCR
q_igf1_R	CAGCCTCCTTAGATCACAGCTC	qPCR
q_fgf2_F	AGCGGCTGTACTGCAAAAACGG	qPCR
q_fgf2_R	CCTTTGATAGACACAACTCCTCTC	qPCR
q_eif3i_F	CAGAACGTCCTGTCAACTCAGC	qPCR
q_eif3i_R	CTTGCCAATCCTGGTGGAGGTT	qPCR
q_cas_F	AACCTATGCCCACCTGTTCG	qPCR
q_cas_R	AGGATTGTCTTGCCGGACTG	qPCR
q_ms2_F	CTGGGAGAGGGCTCCTACTT	qPCR
q_ms2_R	TCATGGTTGGGCCAGGATTC	qPCR
gapdhF	GTCTCCTCTGACTTCAACAGCG	qPCR
gapdhR	ACCACCCTGTTGCTGTAGCCAA	qPCR

**Fig. 1:**
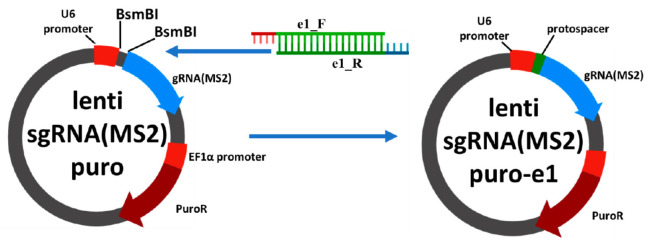
Schematic cloning of lenti sgRNA(MS2)_puro series plasmids. To obtain a vector encoding chimeric guide RNA, the lenti sgRNA(MS2)_puro vector was treated with the BsmBI restriction endonuclease and ligated with an oligonucleotide duplex corresponding to one of the protospacer sequences (*Table 1*). Ligation products were cloned using the* E. coli *Top10 strain. The insertion structure was confirmed by sequencing

The Appendix 1 contains Fig. 101 and Table 1

## Appendix 2

**Table 1:** List of plasmids (with full and abbreviated names) for specific activation of the expression of growth factor genes

Plasmid full name	Duplex	Abbreviation	Gene
lenti sgRNA(MS2)_puro-e1	e1_F/e1_R	e1	EIF3I
lenti sgRNA(MS2)_puro-e2	e2_F/e2_R	e2	EIF3I
lenti sgRNA(MS2)_puro-e3	e3_F/e3_R	e3	EIF3I
lenti sgRNA(MS2)_puro-e4	e4_F/e4_R	e4	EIF3I
lenti sgRNA(MS2)_puro-e5	e5_F/e5_R	e5	EIF3I
lenti sgRNA(MS2)_puro-e6	e6_F/e6_R	e6	EIF3I
lenti sgRNA(MS2)_puro-f1	f1_F/f1_R	f1	FGF-2
lenti sgRNA(MS2)_puro-f2	f2_F/f2_R	f2	FGF-2
lenti sgRNA(MS2)_puro-f3	f3_F/f3_R	f3	FGF-2
lenti sgRNA(MS2)_puro-f4	f4_F/f4_R	f4	FGF-2
lenti sgRNA(MS2)_puro-f5	f5_F/f5_R	f5	FGF-2
lenti sgRNA(MS2)_puro-f6	f6_F/f6_R	f6	FGF-2
lenti sgRNA(MS2)_puro-i1	i1_F/i1_R	i1	IGF-1
lenti sgRNA(MS2)_puro-i2	i2_F/i2_R	i2	IGF-1
lenti sgRNA(MS2)_puro-i3	i3_F/i3_R	i3	IGF-1
lenti sgRNA(MS2)_puro-i4	i4_F/i4_R	i4	IGF-1
lenti sgRNA(MS2)_puro-i5	i5_F/i5_R	i5	IGF-1
lenti sgRNA(MS2)_puro-i6	i6_F/i6_R	i6	IGF-1

Plasmids for specific activation of the expression of growth factor genes were constructed based on the lenti sgRNA( MS2)_puro vector (Addgene #73795). To obtain a vector encoding chimeric guide RNA, the lenti sgRNA(MS2)_ puro vector was treated with BsmBI restriction endonuclease and ligated with an oligonucleotide duplex (the primer sequences are provided in Appendix 1) corresponding to one of the protospacer sequences.

The Appendix 2 contains Table 201

## Abbreviations

FBS: fetal bovine serum
HEK293: human embryonic kidney 293 cell line
IGF-1: insulin- like growth factor 1
FGF-2: fibroblast growth factor 2
EIF3I: eukaryotic translation initiation factor 3 subunit I
SAM: synergistic activation mediator
sgRNA: single-guide RNA
